# Pushing the limits of crystallography

**DOI:** 10.1107/S160057671601637X

**Published:** 2016-11-18

**Authors:** Janusz Wolny, Ireneusz Buganski, Pawel Kuczera, Radoslaw Strzalka

**Affiliations:** a Faculty of Physics and Applied Computer Science, AGH University of Science and Technology, Krakow, Poland

**Keywords:** Debye–Waller correction, phasons, quasicrystals, statistical methods, average unit cell

## Abstract

A very serious concern of scientists dealing with crystal structure refinement, including theoretical research, pertains to the characteristic bias in calculated *versus* measured diffraction intensities, observed particularly in the weak reflection regime. This bias is here attributed to corrective factors for phonons and, even more distinctly, phasons, and credible proof supporting this assumption is given. The lack of a consistent theory of phasons in quasicrystals significantly contributes to this characteristic bias. It is shown that the most commonly used exponential Debye–Waller factor for phasons fails in the case of quasicrystals, and a novel method of calculating the correction factor within a statistical approach is proposed. The results obtained for model quasiperiodic systems show that phasonic perturbations can be successfully described and refinement fits of high quality are achievable. The standard Debye–Waller factor for phonons works equally well for periodic and quasiperiodic crystals, and it is only in the last steps of a refinement that different correction functions need to be applied to improve the fit quality.

## Abstract 2

A statistical method is discussed, which solves the problem of bias in fitted *versus* observed diffraction intensities for quasicrystals.

## 1. Introduction   

Crystallography deals with the solution and refinement of structures based on their diffraction patterns. In recent years, it has been evident that the theoretical description of X-ray diffraction by crystals is still of scientific concern. Novel refinement strategies are still being sought and developed to improve the accuracy of solved structures. *Ab initio* phase measurement (Weckert & Hümmer, 1997 ▸), initially considered as an ineffective tool for structure solution mainly because of the low precision of the measurement (Shen, 2003 ▸), was subsequently shown to be useful for improving the structural resolution of NH_4_H_2_PO_4_ and an experimental strategy for future application was proposed (Morelhão *et al.*, 2015 ▸). The kinematical theory of diffraction was shown to be valid not only for the ‘imperfect crystal’ model proposed by Darwin (1922 ▸) but also for distorted crystals (Fewster, 2016 ▸) for which the reduction of dynamical effects is significant. A diffraction experiment requires a crystalline sample (Ooi, 2010 ▸), but simulations performed on helical structures with twisted waves show the existence of constructive/destructive interference even for noncrystalline samples as long as the wave propagation and the molecules are axially aligned (Jüstel *et al.*, 2016 ▸). Another promising method for structure determination of molecular samples requires porous media to impose crystalline order (Inokuma *et al.*, 2013 ▸). The sample need not be crystalline and trace amounts are sufficient for experimental treatment. To solve highly complex structures (*e.g.* proteins, quasicrystals, complex metallic alloys), high-quality diffraction patterns are a must. The role of weak reflections is of extreme importance. The information contained in a weak reflection is of equal interest to that contained in a strong reflection. From the experimental point of view, using synchrotron radiation, it is possible to measure diffraction intensities with a dynamic range of seven or more orders of magnitude, and even powder diffraction structure factors can be measured with high accuracy (Nishibori *et al.*, 2007 ▸; Wahlberg *et al.*, 2016 ▸) comparable to the dynamical *Pendellösung* method (Kato, 1988 ▸). To overcome the detector limitations it is also possible to combine datasets with different exposure times per frame or different primary beam intensities (Kuczera *et al.*, 2012 ▸, 2014 ▸). The problem lies primarily in appropriate data treatment and structure refinement. Any measured diffraction pattern is modified by factors related to the perturbations of ‘idealized’ atom positions. A commonly used correction term is the Debye–Waller factor (Debye, 1914 ▸; Waller, 1923 ▸), which takes into account the thermal excitations of the crystal lattice. In the simplest picture, for the sake of calculating the correction, atoms are considered to vibrate independently in a mean potential (Bürgi, 1995 ▸). Isotropic and anisotropic distributions of motion are used in practice. Restrictions on the symmetry of the anisotropic displacement tensor were developed by Thorkildsen & Larsen (2015 ▸). A recently proposed refinement procedure based on the lattice dynamical model takes into account concerted atomic motion to properly describe the peak intensity correction (Hoser & Madsen, 2016 ▸). Although the quality of the correction is similar to that obtained using anisotropic displacement parameters, this theory cannot be directly applied to the complex structures of quasicrystals (Shechtman *et al.*, 1984 ▸; Dotera *et al.*, 2014 ▸; Engel *et al.*, 2014 ▸; Cockayne *et al.*, 2016 ▸; Förster *et al.*, 2013 ▸; Takakura *et al.*, 2007 ▸) owing to the lack of periodicity and the resulting complex atomic motion (de Boissieu, 2011 ▸, 2012 ▸). To account for the latter, a mean field approximation is the only alternative. The generalized Debye–Waller factor (Bancel, 1989 ▸; Lubensky *et al.*, 1986 ▸) can be written as 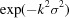, where *k* is the scattering vector magnitude in physical or perpendicular space and 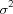 is the variance of the statistical distribution of atoms in physical or perpendicular space (sometimes also called inner space or phasonic space). The above formula is correct only for Gaussian distributions. As far as the phononic contribution is concerned, the approximation made by the Gaussian function is justified only for small atomic displacements from the equilibrium position. On the other hand, the description of the influence of phasonic disorder on the peaks’ intensities fails significantly in the small-peak regime (Buganski *et al.*, 2016 ▸). The Debye–Waller correction with respect to phasons can be made exclusively for a random tiling type of structure (Henley *et al.*, 2001 ▸; I. Buganski, R. Strzalka & J. Wolny, unpublished). The Debye–Waller factor in generalized form can also be used to calculate the intensity of a diffraction peak related to a statistical distribution of atomic positions with respect to a reference lattice (Wolny, 1992 ▸, 1993 ▸; Wolny & Kozakowski, 2003 ▸).

In this work, we show the limitations of the standard Debye–Waller correction (exponential form) in terms of handling the phasonic disorder. We focus on quenched phasons (static or frozen phasons) appearing as atomic flips in the quasicrystalline structure with no dynamical considerations. Two motivations underlie our study. The first is to include weak reflections in the refinement process. The modern diffraction experiment enables us to collect data with a wide range of intensities, but the refinement results show strong bias in the small-peak regime in the plot of fitted *versus* observed intensities. We attribute this fact to the improper use of the exponential Debye–Waller factor for correction for phasons. Secondly, the exponential phasonic correction term is proved to be fully valid in the random-tiling approximation but there is no proof of its correctness and physical soundness in the general case. Our study leads to a new type of structure factor formula. Weak reflections that were underestimated by the previous refinement strategy can now be properly included in the structure refinement. In this paper we also investigate the pertinence of the Debye–Waller correction for phonons. Even though the Debye–Waller correction for phonons is applicable for quasicrystalline structures as well, which we prove in this paper, we suggest testing other functions as a small improvement can be observed for peaks with large scattering vector. The calculations are performed for vertex decoration models based on a one-dimensional Fibonacci chain and two-dimensional Penrose tiling. Neither the model selection nor its dimensionality have any impact on the generality of the results presented here, which can be used for refinement of any structure (periodic crystals, quasicrystals, defected structures and any type of disordered systems). The choice of the Fibonacci chain as a model allows us to easily analyze the influence of both physical and perpendicular space fluctuations on the diffraction pattern. The results obtained for Penrose tiling are of significant practical importance.

## 2. AUC method   

**Figure 1:**
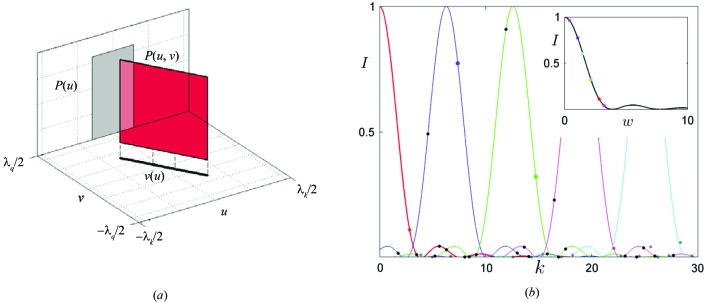
(*a*) The probability distribution 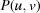 for an ideal Fibonacci chain. The marginal distribution 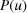 is presented along with the TAU2 scaling. (*b*) The diffraction pattern of the ideal Fibonacci chain with the envelopes of the first five peaks marked. In the inset the diffraction pattern reduced to a single envelope is shown, where 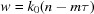 and 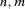 are the peaks’ indices. Highlighted peaks from a full pattern are shown in the reduced pattern with the same markings.

The structure factor used in this paper is calculated in a mathematically strict way, as a Fourier transform of a probability distribution of atoms calculated with respect to the reference lattice (Wolny, 1998 ▸). For a selected scattering vector 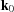 we construct a hypothetical reference lattice with a period of 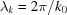. The positions of the atoms with respect to the reference lattice are denoted as *u* (obviously 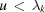). The bounded distribution of *u*, denoted as 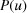, is called the average unit cell (AUC). A Fourier transform of 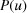 correctly describes the structure factor for any integer multiple of 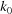 (Wolny *et al.*, 2014 ▸, 2015 ▸). Therefore, for each distribution 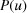, there exists a periodic series of diffraction peaks (see Fig. 1 ▸ *b*). In the case of incommensurately modulated structures or quasicrystals another scattering vector is necessary, 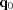, also called the modulation vector or satellite peaks vector. Using two reference lattices, with periods 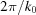 and 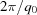, we can define a probability distribution 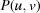. The variable *u* defines the atom position in the first reference lattice and the variable *v* that in the second. It can be easily shown that the structure factor for a scattering vector written as 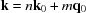 is an 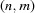-mode Fourier transform of the distribution 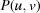 (Buczek *et al.*, 2005 ▸). For incommensurately modulated structures as well as for quasicrystals, the distribution 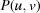 is nonzero only along certain curves. In particular, for quasicrystals scaled with the golden mean ratio τ, these are linear segments with the slope of 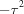 (so-called TAU2 scaling). The distribution 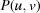, as well as its trace in the 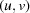 domain (TAU2 scaling) and its marginal distribution 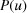, is depicted in Fig. 1 ▸(*a*). For a harmonically modulated structure the scaling relation is more complicated. Details of the scaling relation and AUC construction for a harmonically modulated structure are discussed by Wolny *et al.* (2016 ▸). If the scaling property is used, the diffraction pattern calculation for a Fibonacci chain can be reduced to a one-dimensional Fourier transform (Wolny *et al.*, 2015 ▸). The marginal distribution 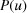 [for the Fibonacci chain shown in Fig. 1 ▸(*a*)] of the full distribution 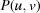 is enough to describe the whole diffraction pattern of the structure.

Knowledge of 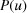 is sufficient to calculate a full one-dimensional diffraction pattern of any quasicrystal. In the higher-dimensional (*n*D) space approach (de Bruijn, 1981 ▸; Yamamoto, 1996 ▸; Steurer & Deloudi, 2009 ▸; Janssen & Janner, 2014 ▸), the distribution 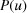 is related to the so-called atomic surface in perpendicular space. It can be easily shown (Wolny *et al.*, 2002 ▸) that in the *n*D approach the AUC is an oblique projection of the atomic surface on the physical space. The projection direction is perpendicular to the *n*D reciprocal space vector. Therefore, the choice of *n*D reciprocal space vector fully defines the distribution 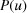 used for the diffraction pattern calculation.

## 3. Debye–Waller factor for periodic crystals   

**Figure 2:**
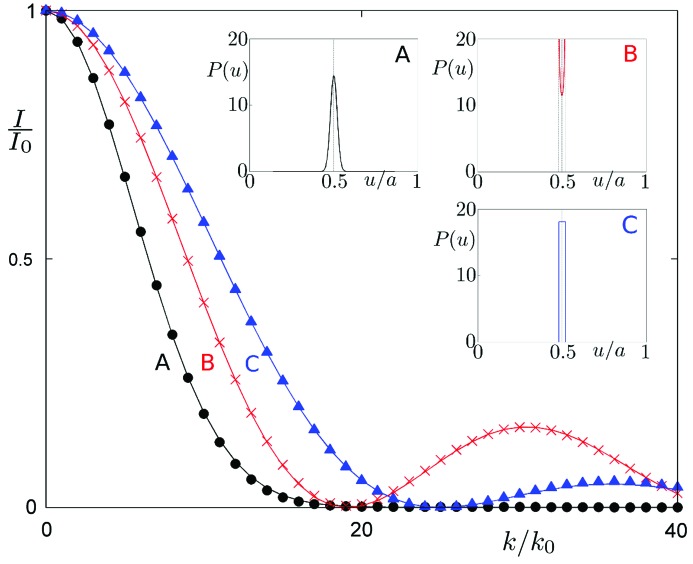
Phonons in periodic crystals. Envelope functions of the diffraction peaks for a periodic crystal with phononic disorder obtained by three different shapes of atomic distributions around ideal positions: Gaussian (black circles, curve A), Bessel function (red crosses, curve B) and cardinal sine function 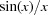 (blue triangles, curve C). The diffraction pattern is periodic with a discrete set of peaks – note the equidistantly distributed peaks. The corresponding probability distributions 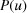 for a periodic structure given by (*a*) Gaussian, (*b*) harmonic and (*c*) uniform distributions are shown in insets. For ideal positions the Dirac delta function applies (marked with dashed vertical lines in each inset). The periodic cell parameter is denoted as *a*. The envelope function is the square of the Fourier transform of the corresponding distribution function 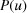.

It is possible to construct the AUC for periodic crystals – it is equivalent to the traditional unit cell. For an ideal periodic structure the probability distribution 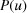 is given by a Dirac delta function centered at some value 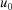. The Fourier transform of the Dirac delta function is constant over the whole range of the scattering vector. The diffraction pattern consists of a periodic series of peaks with periodicity of 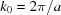, where *a* is the lattice constant, as shown in Fig. 2 ▸. We used a relative peak intensity, *i.e.* the ratio of intensity 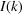 with thermal fluctuations and idealized intensity (no thermal motion) 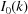. Only peaks with 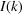 > 10^−2^ in a scale reduced to 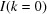 are shown. For a real structure the structure factor is given as a Fourier transform of the probability distribution 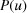 depicted in Fig. 2 ▸ (insets). For a Gaussian probability distribution, 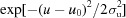, the structure factor is also a Gaussian function with a standard deviation 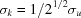. Therefore the envelope function of the diffraction peaks is given by 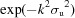, as shown in Fig. 2 ▸. The envelope function gives the peak intensities at the positions described by integer multiples of 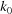. The above formula is known as the Debye–Waller factor and is related to the atom fluctuations in physical space (due to the thermal motion; Trueblood *et al.*, 1996 ▸). It is important to emphasize that the Debye–Waller factor in the form of a Gaussian function properly describes the reduction in peak intensities only for thermal fluctuations given by a Gaussian distribution. For other distributions a Debye–Waller factor written as a second moment of the distribution 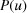 is used. The quality of such an approximation decreases for larger values of the argument 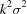. The impact of the approximation on the diffraction pattern is depicted in Fig. 2 ▸. For a Gaussian distribution we get the classical form of the Debye–Waller factor. A uniform distribution is better described by a 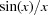 factor. Harmonic distributions, on the other hand, are best described by Bessel functions. In each case the amplitude of thermal vibrations of atoms is given by 2% of the average distance between atoms (for periodic crystals this is the cell parameter *a*). It can be said that for the considered cases the classical Debye–Waller factor works well for small corrections, whereas it performs much worse for the strong ones. Only for Gaussian distributions does the use of the classical Debye–Waller factor correctly describe the diffraction pattern. In any other case it is an approximation, which gets worse as the intensity correction increases.

## 4. Model structure – Fibonacci chain   

**Figure 3:**
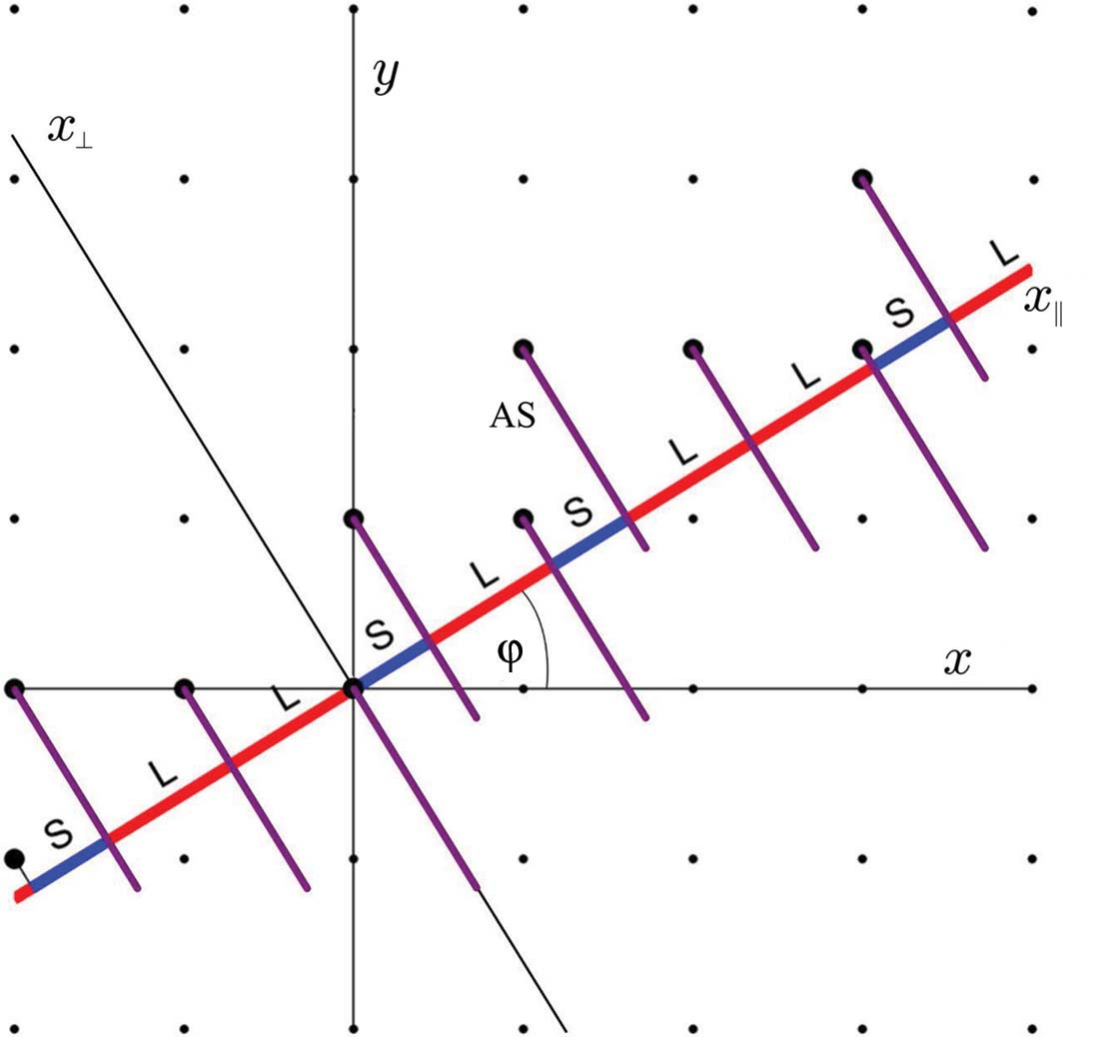
The higher-dimensional description of the Fibonacci chain. Nodes arranged in 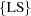 sequence are obtained by a cut of the segment lines, called the atomic surface (AS), attached to the vertices of the square lattice (*x*, *y*) with axis 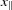. Direction 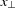 spans the perpendicular space where the ASs are spanned. The slope of 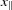 with respect to *x* is defined as 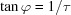.

The Fibonacci chain is a model example of a one-dimensional aperiodic (quasiperiodic) structure (Senechal, 1995 ▸; Baake & Grimm, 2013 ▸). In one-dimensional physical space the Fibonacci chain can be generated using the following recursion rules: 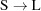; 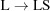. In a single step every short distance (S) becomes a long distance (L) and every long distance (L) transforms to a long–short pair (LS). It is possible to use the *n*D method presented in Fig. 3 ▸ for a description of quasicrystals. For the Fibonacci chain we use a two-dimensional square lattice. We place a so-called atomic surface (AS) at every lattice node. The physical space is chosen in an incommensurate direction in the two-dimensional square lattice. In the case of the Fibonacci chain the slope of the physical space direction is equal to 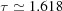. This guarantees aperiodicity. The AUC probability distribution for a Fibonacci chain is shown in Fig. 1 ▸(*a*). It is a uniform distribution with a width related to the reference lattice period (Wolny, 1998 ▸). Such a probability distribution can also be understood as an oblique projection of the atomic surface (Kozakowski & Wolny, 2010 ▸). The diffraction pattern can be calculated as a Fourier transform of either the AS in perpendicular space or the AUC distribution in physical space (Strzalka *et al.*, 2015 ▸). The two methods are absolutely equivalent. In the case of the AUC, calculations can be carried out knowing the distributions explicitly or, alternatively, a knowledge of the distribution’s moments is also sufficient (Buganski *et al.*, 2016 ▸).

If we allow for flips in the structure of a Fibonacci chain, *i.e.* a point interchange of the form LS 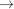 SL, or if during structure generation we randomly use the rule L 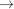 SL instead of L 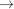 LS we will obtain a defected Fibonacci chain. Such position flips are called phasons (Lipp *et al.*, 2010 ▸), and this kind of sequence formation was experimentally observed, for example, during the formation of nanodomains on the decagonal AlCuCo surface (Duguet *et al.*, 2011 ▸). In the remainder of the article we will try to describe the influence of phonons and phasons on the diffraction pattern by introducing appropriate correction terms for the intensities of diffraction peaks.

In our simulated structure we obtained the effect of phason flips by randomly swapping the neighboring distances L and S in a numerically generated structure of 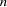 = 70 000 points of a Fibonacci chain. We perform a flip if the value of a standard uniform random variable is greater than 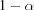, where 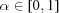. Because each S tile potentially participates in the flip occurrence, the expected value of the number of flips is 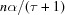. The value in the denominator comes from the fact that the ratio of the number of tiles 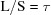, and thus the probability of finding an S tile is equal to 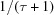.

## 5. Debye–Waller factor for quasicrystals   

**Figure 4:**
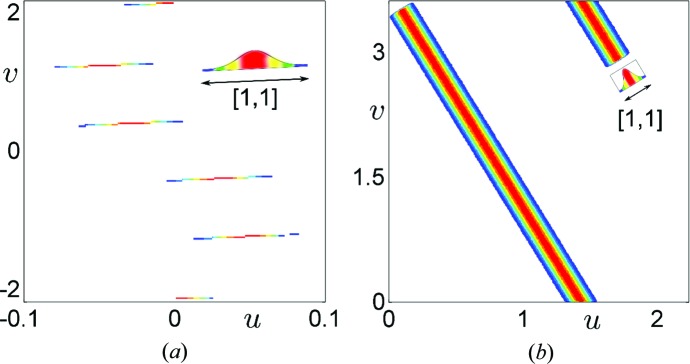
The influence of phonons on the distribution 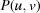 for (*a*) a commensurately modulated crystal, 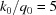, and (*b*) the Fibonacci chain, 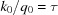. The characteristic TAU2 scaling relation is smeared along the [1, 1] direction in the 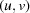 framework. The broadening is caused by Gaussian smearing of each atomic position due to thermal vibrations of atoms. In each case the amplitude of the phonons is 2% of the average distance between atoms in the structure.

**Figure 5:**
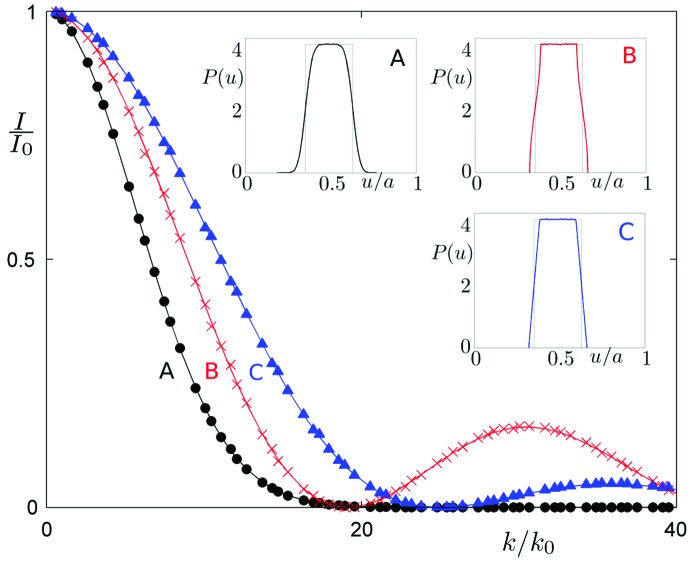
The envelope function and probability distributions 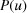 for the Fibonacci chain (one-dimensional quasicrystal) with phonons. The notation from Fig. 1 ▸ has been used. The set of peaks is in general infinitely dense, and its distribution is aperiodic. For clarity only peaks with limited indices and intensities are shown. For ideal positions (no phonons) the uniform probability distribution 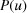 is observed [marked with dashed lines in the insets; compare with Fig. 4 ▸(*a*)]. Reference lattice parameter 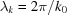.

In physical space the Debye–Waller factor works relatively well for narrow probability distributions, which are characteristic for ideal periodic structures, *i.e.* for periodic crystals. The issue becomes more complicated for quasicrystals. The lack of periodicity broadens the probability distributions even for ideal structures (Wolny, 1998 ▸). The scaling relation 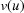 provides the best tool to consider phononic smearing of the probability distribution as it is clearly recognizable even for commensurately modulated structures, which can be considered as periodic (see Fig. 4 ▸). This additional broadening caused by phonons modifies the 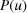 distribution as indicated in Fig. 5 ▸ (insets). We need the full 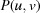 distribution, depicted in Fig. 4 ▸(*b*), to properly describe the diffraction pattern. Gaussian smearing of each atomic position leads to smearing of the characteristic TAU2 scaling. The amplitude of phonons is defined again as 2% of the average distance between atoms (for a Fibonacci chain it is 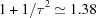). The marginal distributions are shown in Fig. 5 ▸ (insets). The use of the Debye–Waller factor calculated according to the appropriate distribution of position fluctuations yields correct intensities for all diffraction peaks. The diffraction pattern in this case is much denser than the analogous diffraction pattern for a periodic crystal (Fig. 5 ▸). This is because the diffraction pattern of a quasicrystal is theoretically infinitely dense (Levine & Steinhardt, 1986 ▸).

If we compare the diffraction patterns for a periodic crystal and a quasicrystal (Figs. 2 ▸ and 5 ▸) it is clear that for both cases the Debye–Waller factor modifies the peak intensities in the exact same manner. There are no major differences for a practical use of the phononic Debye–Waller factor for quasicrystals and periodic crystals (see Appendix *A* [App appa]). The choice of the right distribution of position fluctuation with respect to the idealized structure is a major issue. This can be done by trial and error, based on the quality of the obtained fit.

**Figure 6:**
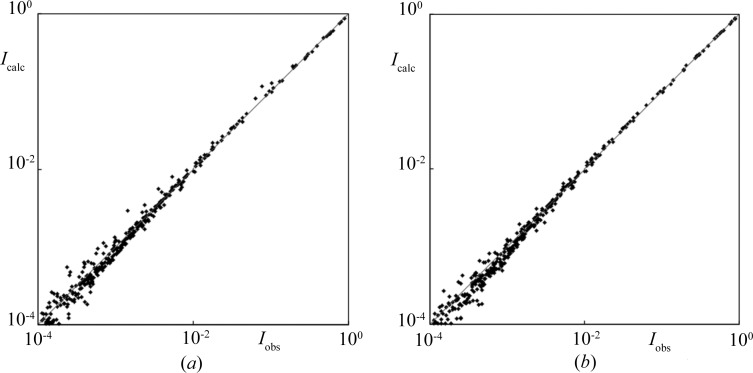
Calculated (*I*_calc_) *versus* numerically simulated (*I*_obs_) diffraction intensities for the Fibonacci chain with phonons and the Debye–Waller correction in the form of (*a*) a Gaussian (standard exponential Debye–Waller factor) and (*b*) a Bessel function. The fit agreement is at a level of 3% (Gaussian) and 1.8% (Bessel function).

In Fig. 6 ▸ we show a comparison of simulated and fitted diffraction patterns for the Fibonacci chain. For the simulated structure the atomic positions were modified by fluctuations expressed by a sine function. Then, an idealized diffraction pattern was modified by the traditional Debye–Waller factor and fitted to the simulated pattern. The agreement factor, understood as the conventional *R* factor (Prince, 2004 ▸), is 3%. If the Gaussian distribution of the Debye–Waller factor is changed to a Bessel function, the agreement factor is equal to 1.8%. In this case the improvement is caused by the use of a correction in the form of a Bessel function.

Generally, for scalable structures, the probability distributions can be lifted to higher dimensions. This leads to the so-called atomic surface shown in Fig. 3 ▸. It is possible to use a higher-dimensional periodic space for the description of such structures. The ASs are placed at the nodes of the higher-dimensional space. For the simplest quasiperiodic structures, like the one-dimensional Fibonacci chain, two-dimensional Penrose tiling or three-dimensional primitive icosahedral Ammann tiling, the higher-dimensional lattice is regular and the ASs are simple geometric shapes (a line section, set of four pentagons and a rhombic triacontahedron, respectively). The diffraction patterns can be calculated as Fourier transforms of these ASs for a suitable perpendicular component of the scattering vector. However, it must be emphasized that such methodology works for defect-free structures. The influence of phonons on the probability distributions is solely a physical space phenomenon. Therefore, the shape of the ASs, as part of the perpendicular space, remains unchanged under phononic disorder. The statistical method allows for incorporating phononic disorder into a structural analysis of quasiperiodic structures within one unified approach. The full mathematical justification is presented in Appendix *A* [App appa].

## 6. Phasonic Debye–Waller factor   

**Figure 7:**
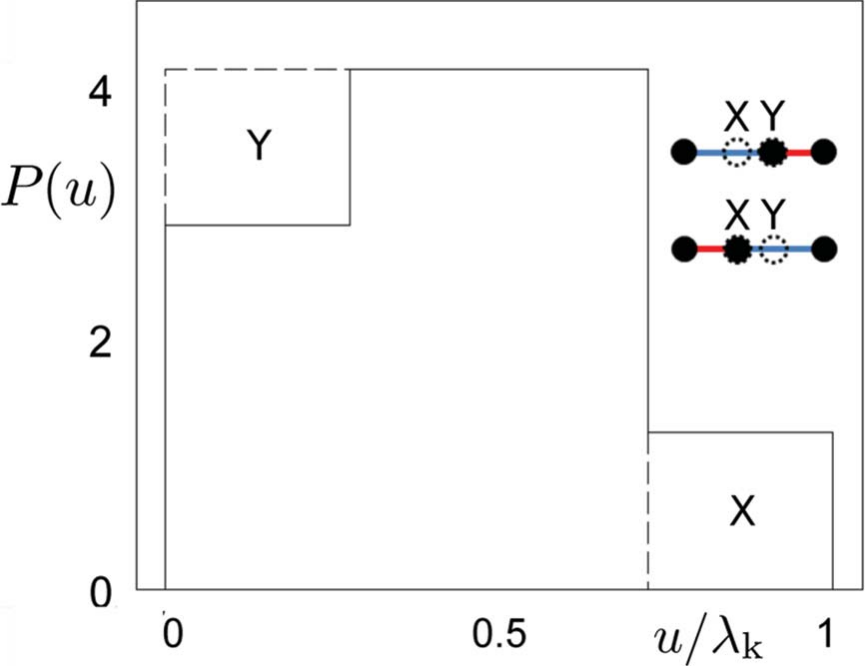
Probability distribution for the Fibonacci chain under phasons. The distribution for the ideal Fibonacci chain is drawn with dashed lines. Part of a distribution X, related to the mid position in the sequence LS for the ideal chain (circle with dotted circumference), is shifted to a new position Y. The area of the moved block is proportional to α with respect to the area of full distribution for the ideal chain, where α is the flip probability.

**Figure 8:**
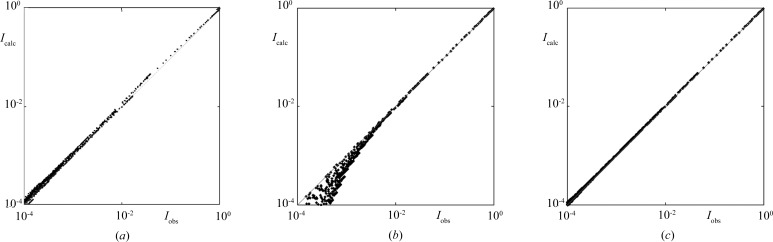
Calculated (*I*_calc_) *versus* numerically simulated (*I*_obs_) diffraction intensities for the Fibonacci chain with phasons (*a*) and after phasonic correction in the form of (*b*) the multiplicative standard Debye–Waller factor or (*c*) the additive term (sum of Bessel functions). The phason occurrence probability is 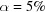. The standard phasonic Debye–Waller factor used in a refinement procedure favors strong reflections, for which a clear mismatch is visible in (*a*). This is the reason why the fit for weak reflections is completely wrong in graph (*b*). The statistical approach does not cause these effects.

Contemporary crystallography still struggles to find a way to describe structures with correlated disorder (Keen & Goodwin, 2015 ▸). A perfect example is the phason flip where a group of atoms rearrange their positions in a specific manner imposed by geometric restrictions (Engel & Trebin, 2008 ▸). It is believed that phasons play a crucial role in the stabilization mechanism of quasicrystals (Janssen & Janner, 2014 ▸; Kiselev *et al.*, 2012 ▸). Therefore, an appropriate treatment of diffraction data with respect to phasons is required. The growth of a quasicrystal itself occurs because of reconstruction of atomic positions through phason flips (Nagao *et al.*, 2015 ▸), but there exists also a theoretical possibility of perfect quasicrystal formation (Achim *et al.*, 2014 ▸). Phason flips occurring in the structure of quasicrystals are usually randomly distributed with a small probability of collective phason formation (Engel *et al.*, 2010 ▸). In the case of phason flips and their statistical description within the AUC concept, part of the distribution is shifted from one place to another (Fig. 7 ▸). Usually, this leads not only to a broadening of the probability distribution but also to its fragmentation. In the most popular approach, the influence of phasons on the diffraction pattern is described in the perpendicular space. A Gaussian approximation of the form 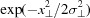 leads to a Debye–Waller factor of the form 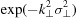, where 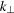 is the perpendicular component of the scattering vector. Frequently, 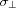 is a fit parameter. It is worthwhile remembering that it is only a phenomenological parameter and that the Debye–Waller factor in the form mentioned above only describes a Gaussian distribution approximation valid in the case of random tiling. For quasicrystals described by aperiodic tiling with phason disorder, such an approximation breaks down for weak reflections, which are actually in a majority (Fig. 8 ▸ *a*). Replacing the underlying Gaussian distribution assumption with a more suitable one, considering cuts in the AUC, yields much better results (Fig. 8 ▸ *c*). The use of the ‘classical’ Debye–Waller factor leads to the characteristic bias for weak reflections shown in Fig. 8 ▸(*b*). Only the use of a sum of cardinal sine functions as an underlying distribution for a Debye–Waller-like correction term radically improves this problem and enables the practical use of weak reflections in the refinement process. We emphasize that for aperiodic structures, and especially for quasicrystals, the number of weak reflections is far greater than that of strong ones. The use of weak reflections in the refinement process is therefore of fundamental importance. In our approach, we exchange the multiplicative classical Debye–Waller factor for an additive factor based on a sum of first-order spherical Bessel functions of rank 0. This step is theoretically justified (see Figs. 7 ▸ and 9 ▸ and Appendix *B* [App appb]). It is also important to stress that the characteristic bias in our model calculations is observed in modern refinement results which make use of the standard phasonic Debye–Waller factor. As may be easily checked from real experimental data (Kuczera *et al.*, 2012 ▸, 2014 ▸), the bias is significantly larger for reflections with larger 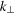 values. This observation independently confirms our hypothesis.

## 7. Model structure – Penrose tiling   

The two-dimensional quasilattice can be obtained by Penrose tiling, where the nodes are spanned by two kinds of rhombuses: thick and thin, with their volume ratio given by τ. In physical space the tiling can be obtained by applying the so-called matching rules defining the mode of tile assembly to fill the plane with no holes or overlaps. Penrose tiling is widely used for the construction of a starting model for decagonal quasicrystals (Steurer & Deloudi, 2009 ▸; Kozakowski & Wolny, 2010 ▸; Takakura *et al.*, 2001 ▸). The AUC shape constructed within the statistical method takes the form of four pentagons, two smaller and two larger ones. In the *n*D approach the four pentagons decorate a four-dimensional hyperrhombohedron, which is a four-dimensional unit cell for decagonal quasicrystals. The structure factor for Penrose tiling can be expressed in the statistical method simply as a Fourier transform of the marginal distribution 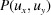, which is now a two-dimensional object, since the TAU2 scaling property is the same as for the Fibonacci chain (Wolny *et al.*, 2002 ▸; Kozakowski & Wolny, 2010 ▸).

**Figure 9:**
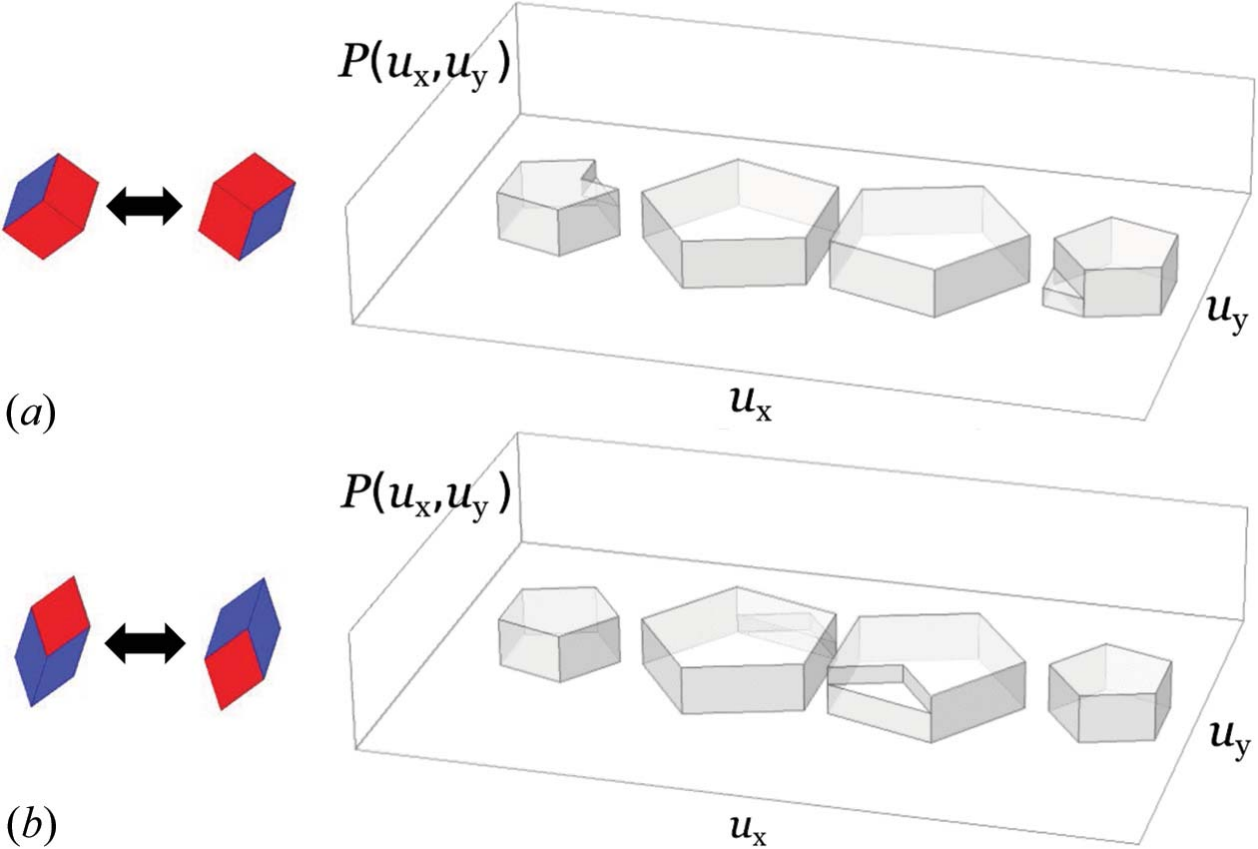
Distribution 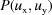 for Penrose tiling obtained for different types of phason flips: (*a*) thick–thick–thin and (*b*) thin–thin–thick tile configurations in a hexagon. After phason flips the ideal four pentagons are changed and some triangular parts are shifted to new locations, depending on the type of flip.

A phason flip in the case of Penrose tiling will be understood as a rearrangement of tiles such that groups of two thick and one thin rhombuses swap their positions at a certain orientation in physical space. Another possibility is the swapping of tiles in a group of two thin and one thick rhombuses. For a better understanding, the two kinds of flips are shown in Fig. 9 ▸. The rearrangement of tiles can be effectively modeled as a small shift of the inner vertex of the group of three rhombuses. The same is true in the case of the Fibonacci chain (see Fig. 7 ▸). Our calculations are performed for a structure consisting of nearly 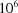 nodes and different probability ratios of flip occurrence, α.

## 8. Phasons for Penrose tiling   

**Figure 10:**
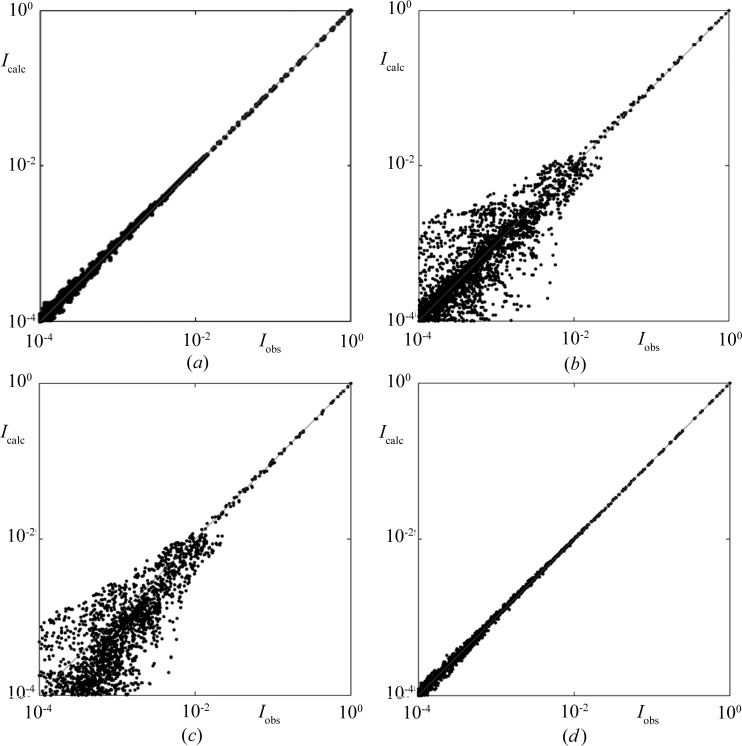
Correlation plots of calculated (*I*_calc_) *versus* numerically simulated (*I*_obs_) diffraction intensities for Penrose tiling: (*a*) ideal (no phasons) and (*b*)–(*d*) with phasons. The slight smearing of points along the line in (*a*) is caused by the finite size of the sample only. The probability of phason occurrence is 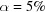. No correction factor was used in (*b*), and phasonic correction in the form of the multiplicative standard Debye–Waller factor and the additive term (sum of Bessel functions) was applied to (*c*) and (*d*), respectively. The correlation in (*d*) is almost perfect, with small deviations caused by the finite size of the sample [*cf.* (*a*)].

As in the case of the Fibonacci chain, for Penrose tiling phasons also cause a fragmentation of the probability distribution and some triangular parts are shifted to positions that do not constitute ideal tiling (Fig. 9 ▸). The diffraction pattern of the disordered structure can again be calculated in two ways: as a Fourier integral over all atomic positions (treated as the observed dataset) or using the structure factor fitted to the data points with a possible correction for phasons. In Fig. 10 ▸(*a*) a log–log plot of the fitted *versus* observed intensities is shown for the ideal Penrose tiling with no phason flips. The small deviation from a straight line is caused only by the finite size of the sample. If phasons are introduced into the structure, the picture looks very different. Depending on the degree of phasonic disorder the *I*_calc_ *versus I*_obs_ line gets broadened and a strong dispersion of peaks is observed, particularly in the weak reflection regime, which is clearly seen in Fig. 10 ▸(*b*). The Debye–Waller correction for phasons can now be calculated in Gaussian form again, but such an approximation does not describe all diffraction peaks correctly, as was the case for the Fibonacci chain. In the log–log plot of fitted *versus* (simulated) observed intensities a strong bias in the weak reflection regime is apparent (Fig. 10 ▸ *c*). At the same time, the spread of data points along the straight line only slightly diminishes and, moreover, the fit becomes worse owing to the systematic deviation. This behavior is a well known fact in modern refinements of quasicrystalline structures (Kuczera *et al.*, 2010 ▸, 2011 ▸, 2012 ▸; Takakura *et al.*, 2007 ▸). The models using the standard phasonic Debye–Waller correction suffer from the improperly treated phasonic disorder.

The statistical method gives a perfect tool to handle phasons. Since we know how phasons affect the probability distributions, and how the structure factor is strictly related to those, the correction for phasons arises naturally. From Fig. 9 ▸ we conclude that the probability distribution obtained for a Penrose tiling with phasons differs from the ideal one by the shifting apart of some triangular components. By restoring the initial distribution, *i.e.* moving the triangular parts back to their initial positions, the calculations of diffraction intensities can be performed in the same way as for a perfect Penrose tiling. Another approach is also possible. The Fourier-transformed triangular parts give an additive factor to the structure factor, as was the case for the Fibonacci chain with phasons. There, the cardinal sine functions emerge from the Fourier transformation of the flat parts of the distribution shifted to new positions. The result of application of the above-mentioned approach to phasons in the Penrose tiling is shown in Fig. 10 ▸(*d*). All peaks are equally well corrected and aligned perfectly on a straight line. No bias is observed and the fit is of the same quality as for ideal Penrose tiling without phasons.

## 9. Summary   

It is possible to fully reconstruct the diffraction pattern using a probability distribution of atoms in the reference lattice. For practical calculations it is sufficient to know the moments of the distribution. However, the number of moments required grows with the degree of complication of the distribution. For a Gaussian distribution knowledge of the second moment is sufficient, and the classical Debye–Waller factor is the result. In the general case the classical Debye–Waller factor is only an acceptable approximation for small corrections and it breaks down for larger corrections.

The Debye–Waller factor has two components: phononic and phasonic. The first is related to thermal motion of atoms in the (quasi)crystal structure, the other to the atomic jumps (flips, phasons). Usually, the phononic part is described in physical space and the phasonic part in the higher-dimensional perpendicular space.

Both components influence the distribution 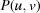. Phonons cause a small broadening of the line along the [1, 1] direction in the 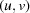 reference system describing the scaling properties (the TAU2 scaling for quasicrystals based on τ; Fig. 5 ▸). Only Gaussian broadening leads to the classical Deby–Waller factor in reciprocal space. This factor is independent of the type of the underlying structure. It is the same for all structures from periodic crystals to quasicrystals. The correct intensity reducing term depends not on the kind of structure but only on the kind of position modulation. In this paper we have presented model calculations for the distributions of atoms described by normal, uniform and harmonic distributions. For each case the use of an appropriate function is necessary to account for the intensity correction. Only the use of an appropriate correction term allows one to refine the structure against the whole measured range of diffraction intensities. To summarize, the classical phononic Debye–Waller factor works equally well for periodic crystals and for quasicrystals; however, the possible improvement of a refinement result can be obtained by trying different correction terms, *e.g.* in the last steps of the refinement procedures.

Completely different conclusions apply to phasonic correction. The standard (Gaussian) correction term fails in the case of quasicrystals and a new approach is needed. Our calculations for model systems – Fibonacci chain and Penrose tiling – show the problem with intensities of weak reflections, which is also a fact described in the literature. We are convinced that the characteristic bias in the fitted *versus* simulated (observed) intensities plot is caused by the incorrect Debye–Waller factor form used in the refinement. The commonly used exponential term is wrong. The argument confirming our hypothesis independently of the simulation results is that the bias concerns mostly peaks with a large perpendicular space component of the scattering vector. Such a feature is observed experimentally. If so, the correction term with perpendicular scattering vector in the exponent, as in case of the Debye–Waller factor, must fail. We have proposed a novel method of dealing with phasons, where knowing the probability distribution of atoms in the reference lattice is enough to reconstruct a full diffraction pattern of a structure with possible phason disorder. Phasons significantly influence the probability distribution by its fragmentation. Including this in a definition of structure factor automatically solves the problem of phasons at the very basic level of calculating the diffraction pattern. Neither any multiplicative correction factor nor iterative fitting of parameters in the Gaussian’s exponent is required. The only free parameter to fit would be the number of phason flips, which has a physical basis. Moreover, by using a statistical method in the refinement of structures with phason flips, very good results are achieved.

The statistical method offers the possibility to retrieve probability distributions directly from diffraction patterns (Kozakowski & Wolny, 2013 ▸). The distributions can also be modeled by some approximation techniques. One of the possible ways is to approximate the shape of the probability distribution by its histograms. This yields a correction factor described by a sum of cardinal sine functions (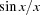). Another approach is to use the Taylor expansion of an envelope function for the moments, where an envelope function is considered a characteristic function of probability distribution. It is also possible to use projections of distributions along certain directions, which leads to a corresponding sum of components.

In conclusion, the appropriate choice of correction factor for phonons and phasons allows the inclusion of very weak reflections of the diffraction pattern in the refinement process of the atomic structure. It is of a great importance for such complex structures as quasicrystals or complex metallic alloys.

## Appendix A

In order to derive the formula for the structure factor with a phononic contribution for a quasicrystal within the statistical approach based on the mean field approximation, we first introduce atomic coordinates ,  in the reference lattice framework for a given position *r*:

(*n* being an integer). The coordinates  can be written as

where ,  denote coordinates without phononic displacement and ,  denote the displacement from the equilibrium position. The distribution  is equal to the convolution of the distribution  and distribution  (pairs *u*,  and  are independent):

The Dirac delta function  is nonzero if displacements  and  are equal. As mentioned, the structure factor is a Fourier transform of the atomic position distribution:

where *h*_1_ and *h*_2_ are diffraction peak indices (integers). Using formula (3) for the distribution  we can reformulate (4) as follows:

If we apply the coordinate change

equation (5) takes the form

The Jacobian of the transformation (6) is equal to 1. According to Fubini’s theorem we can reshuffle the order of integration in equation (7):

The first integral in (8) describes the structure factor without the phononic contribution, and the second gives the phononic correction factor. No assumptions on the displacement distribution were used in the derivation. The result is therefore applicable for any kind of structure type including periodic ones (for ).

## Appendix B

To calculate the structure factor for the Fibonacci chain with phason flips we divide the  distribution into three regions where the distribution is uniform (Fig. 7 ▸). These regions are , ,  in the case of . The heights of the probability density distributions in the respective regions are  and . The coefficient α is the phason flip probability. The structure factor is

Each component  has the following definition:

where

is the amplitude of probability distribution in the *n*th region,

is the width of the *n*th region, and

is the position of the center of the *n*th region.

Equation (10) is the solution of the following integral:

Using the properties of Bessel functions, (10) can be written as

where  denotes the spherical Bessel function of the first kind.
